# Head-to-head intra-individual comparison of biodistribution and tumor uptake of ^68^Ga-FAPI and ^18^F-FDG PET/CT in cancer patients

**DOI:** 10.1007/s00259-021-05307-1

**Published:** 2021-06-17

**Authors:** Frederik L. Giesel, Clemens Kratochwil, Joel Schlittenhardt, Katharina Dendl, Matthias Eiber, Fabian Staudinger, Lukas Kessler, Wolfgang P. Fendler, Thomas Lindner, Stefan A. Koerber, Jens Cardinale, David Sennung, Manuel Roehrich, Juergen Debus, Mike Sathekge, Uwe Haberkorn, Jeremie Calais, Sebastian Serfling, Andreas L. Buck

**Affiliations:** 1 Department of Nuclear Medicine, University Hospital Duesseldorf, Duesseldorf, Germany; 2 Department of Nuclear Medicine, University Hospital Heidelberg, INF 400, 69120 Heidelberg, Germany; 3 Department of Nuclear Medicine, Klinikum rechts der Isar, Technical University of Munich, Munich, Germany; 4 Department of Nuclear Medicine, University of Duisburg-Essen and German Cancer Consortium (DKTK)-University Hospital Essen, Essen, Germany; 5 National Center for Tumor diseases (NCT), Heidelberg, Germany; 6 Clinical Cooperation Unit Radiation Oncology, German Cancer Research Center (DKFZ), Im Neuenheimer Feld 280, 69120 Heidelberg, Germany; 7 Heidelberg Institute of Radiation Oncology (HIRO), Im Neuenheimer Feld 400, 69120 Heidelberg, Germany; 8 Department of Radiation Oncology, University Hospital Heidelberg, Im Neuenheimer Feld 400, 69120 Heidelberg, Germany; 9 Ahmanson Translational Theranostics Division, Department of Molecular and Medical Pharmacology, University of California at Los Angeles, Los Angeles, CA USA; 10 Heidelberg Ion-Beam Therapy Center (HIT), Im Neuenheimer Feld 450, 69120 Heidelberg, Germany; 11 Department of Nuclear Medicine, University of Pretoria and Steve Biko Academic Hospital, Pretoria, South Africa; 12 Clinical Cooperation Unit Nuclear Medicine, German Cancer Research Center (DKFZ), Heidelberg, Germany; 13 Translational Lung Research Center Heidelberg (TLRC), German Center for Lung Research (DZL), Heidelberg, Germany; 14 Department of Nuclear Medicine, University Hospital Würzburg, Würzburg, Germany; 15 Comprehensive Cancer Center Mainfranken, 97080 Wuerzburg, Germany

**Keywords:** FAPI PET/CT, FDG PET/CT, Cancer-associated fibroblast, Various cancer diseases

## Abstract

**Purpose:**

FAPI ligands (fibroblast activation protein inhibitor), a novel class of radiotracers for PET/CT imaging, demonstrated in previous studies rapid and high tumor uptake. The purpose of this study is the head-to-head intra-individual comparison of ^68^Ga-FAPI versus standard-of-care ^18^F-FDG in PET/CT in organ biodistribution and tumor uptake in patients with various cancers.

**Material and Methods:**

This international retrospective multicenter analysis included PET/CT data from 71 patients from 6 centers who underwent both ^68^Ga-FAPI and ^18^F-FDG PET/CT within a median time interval of 10 days (range 1–89 days). Volumes of interest (VOIs) were manually drawn in normal organs and tumor lesions to quantify tracer uptake by SUVmax and SUVmean. Furthermore, tumor-to-background ratios (TBR) were generated (SUVmax tumor/ SUVmax organ).

**Results:**

A total of 71 patients were studied of, which 28 were female and 43 male (median age 60). In 41 of 71 patients, the primary tumor was present. Forty-three of 71 patients exhibited 162 metastatic lesions. ^68^Ga-FAPI uptake in primary tumors and metastases was comparable to ^18^F-FDG in most cases. The SUVmax was significantly lower for ^68^Ga-FAPI than ^18^F-FDG in background tissues such as the brain, oral mucosa, myocardium, blood pool, liver, pancreas, and colon. Thus, ^68^Ga-FAPI TBRs were significantly higher than ^18^F-FDG TBRs in some sites, including liver and bone metastases.

**Conclusion:**

Quantitative tumor uptake is comparable between ^68^Ga-FAPI and ^18^F-FDG, but lower background uptake in most normal organs results in equal or higher TBRs for ^68^Ga-FAPI. Thus, ^68^Ga-FAPI PET/CT may yield improved diagnostic information in various cancers and especially in tumor locations with high physiological ^18^F-FDG uptake.

**Supplementary Information:**

The online version contains supplementary material available at 10.1007/s00259-021-05307-1.

## Introduction

Altered metabolism is one of the hallmarks of cancer [1]. The metabolic switch to utilize glucose (often equated to the Warburg effect) has been successfully exploited by ^18^F-FDG PET imaging since the 1980s. ^18^F-FDG is the most frequently used tracer for oncological PET imaging. However, its known limitations are high uptake in many normal tissues, low glucose transporter and hexokinase activity in certain tumor types, and a lack of specificity [2, 3].

PET imaging of the fibroblast activation protein (FAP) expression in cancer was recently introduced [4, 5]. The first generation of radiolabeled FAP inhibitors (FAPI) is peptidomimetic quinoline derivatives that bind with high affinity to FAP expressed on cancer-associated fibroblasts (CAFs). CAFs are a key component of the tumor stroma or tumor microenvironment. The tumor stroma consists of a variety of benign cells which interact with the tumor cells to promote growth, invasion, and metastasis. However, these cancer-associated cells, which are thought to promote tumor growth, often express unique receptors not found in the same cells outside of the tumor microenvironment. Therefore, an extensive stroma is often associated with metastatic spread and poor prognosis [6, 7].

While many activated CAFs overexpress FAP, ubiquitous resting fibroblasts in healthy tissue have no or low FAP expression. FAP is a type II transmembrane serine protease with post proline dipeptidyl peptidase as well as endopeptidase activity [8]. FAP is overexpressed by stromal fibroblasts in over 90% of epithelial carcinomas. However, FAP expression is not cancer specific: many activated fibroblasts express FAP in wound healing and in nonmalignant diseases such as chronic inflammation, rheumatological diseases, myocardial infarction, lung fibrosis, or liver cirrhosis, among others [8, 9].

The objective of this multicenter retrospective analysis was to perform a head-to-head comparison of ^68^Ga-FAPI to ^18^F-FDG in patients with a variety of tumor types to establish generalizable differences between the two agents. Therefore, we intra-individually compared the biodistribution in normal tissue and tumor uptake of ^68^Ga-FAPI and ^18^F-FDG.

## Methods

### Patients

For this international multicenter retrospective analysis, we screened the database of 6 centers (University Hospital Heidelberg (UKHD, GER), University Hospital Würzburg (UKW, GER), University Hospital Essen (UHE, GER), Technical University Hospital Munich (TUM, GER), University of California Los Angeles (UCLA, USA), University of Pretoria (UP, RSA)) for patients with various cancers who underwent both ^18^F-FDG and ^68^Ga-FAPI PET/CT scans within a maximum of 3 months apart. Seventy-one patients were finally included (UKHD *n* = 29, UKW *n* = 16, UHE *n* = 6, TUM *n* = 1, UCLA *n* = 14, UP *n* = 5). Data were anonymized, centralized (UKHD), and retrospectively analyzed (UKHD) (Supplement Table 1). This present study enhances and evaluates novel aspects of the UKHD patient cohort partially previously described (Ref: 17).

The ^18^F-FDG PET/CT scans were performed as per standard of care for oncologic indications. All patients from German sites gave written informed consent to undergo a ^68^Ga-FAPI PET/CT scan following national regulations, the Declaration of Helsinki, and Good Clinical Practice (GCP). The radiopharmaceutical was produced in accordance with the German Pharmaceuticals Act §13(2b) and the responsible regulatory bodies. The retrospective evaluation of data was approved by the ethics committee of Heidelberg University (permit S016/2018), by the ethics committe from University of Pretoria (881/2019), by the ethics committee of University Hospital Wuerzburg (permit 2021031005), ethics committee of Technical University Hospital Munich (permit 332/21S).

Patients at UCLA were enrolled into the prospective study NCT04147494 conducted under the Radioactive Drug Research Committee (RDRC) program and approved by the institutional review board (IRB#19-000756) and the Jonsson Comprehensive Cancer Center (JCCC) Internal Scientific Peer Review Committee (ISPRC). All UCLA patients gave written informed consent to participate in the parent prospective study. The UCLA IRB approved this anonymized retrospective analysis (approval #20-001663), and the requirement to obtain informed consent was waived.

### PET image acquisition

As this is an international retrospective multicenter study, a variety of PET/CT systems were used for image acquisition and reconstruction (Supplemental Table 2). Overall, whole body images encompassing the patients’ head to mid thighs were obtained for both ^18^F-FDG and ^68^Ga-FAPI scans. For both tracers, PET/CT datasets acquired approximately 1 h after injection were used for analysis. All PET scans were acquired in 3D mode with an acquisition time of 3–5 min/bed position at all sites. The median time interval between ^18^F-FDG PET/CT and ^68^Ga-FAPI PET/CT was 10 days (range 1–89 days). No change of therapy took place in between the scans.

#### ^18^F-FDG imaging

Patients were instructed to fast for at least 6 h before the scan, and blood glucose levels were measured before injection. All patients had serum glucose levels of <150 mg/dl prior to the scan. Median injected activity was 316 MBq (range 233–680 MBq).

#### ^68^Ga-FAPI imaging

Several different ^68^Ga-labeled FAPI ligands of similar chemical composition were used in this study: FAPI-02, *n* = 6; FAPI-04, *n* = 32; FAPI-46, *n* = 32; and FAPI-74, *n* = 1. Radiosynthesis and labeling were performed as described previously [10, 11]. Median injected activity was 185 MBq (range 52–325).

### Image analysis

Volumes of interest (VOI) were placed over the normal organs by one UKHD investigator (JS; supervised by FLG&CK) with a diameter of 1 cm for the small organs (thyroid, parotid gland, myocardium, oral mucosa, and spinal cord) to 2 cm for the other organs (brain, muscle, liver, spleen, kidney, fat, aortic lumen, and lung). Circular regions of interest (ROI) were placed on axial slices around lesions with focally increased tracer uptake and were automatically incorporated into a 3-dimensional volume of interest (ESoft; Siemens). A 40% iso-contouring approach was used for organs as well as lesions. Normal organs and tissue tracer uptake and biodistribution were quantified by SUVmean and SUVmax. Tumor-to-background ratios (TBRs) were determined to quantify the image contrast. The TBR was obtained from the geometric mean of the intra-individual quotients of lesion (SUVmax) to background tissue (SUVmax). TBRs were calculated for metastases in lymph nodes (relative to fat tissue), bone (relative to bone spongiosa), liver (relative to liver parenchyma), and lung (relative to lung parenchyma). In addition, TBRs of all tumors were calculated in relation to different tissue types (blood pool, muscle, fat tissue).

### Statistical analysis

We used descriptive analyses for demographics and tumor characteristics. For description of SUV, arithmetic mean, standard deviation, and median were used. Comparison between ^68^Ga-FAPI- and ^18^F-FDG-SUV in tumor and normal tissue and TBRs was compared with the Wilcoxon signed-rank test. A *p* value of <0.05 was considered statistically significant. All statistical analyses were performed using SPSS Statistics Version 24 (IBM, Armonk, NY, USA), Excel for Mac Version 15.41 (Microsoft, Redmond, Washington, USA) and SigmaPlot 12.5 (Systat Software Inc.) for graphical visualization.

## Results

### Study population

The clinical characteristics of the study population are summarized in Supplement Table 1. The following cancer types were included: head and neck cancer (*n* = 16), lung carcinoma (*n* = 9), biliary-pancreatic cancer (*n* = 12), gastrointestinal tract cancer (*n* = 14, including colon carcinoma, rectal carcinoma, anal carcinoma, cecum carcinoma, sigmoid carcinoma, gastro-esophageal cancer, duodenal cancer), and gynecologic cancer (*n* = 12). The group of other cancers (*n* = 8) consists of a neuroendocrine bladder carcinoma, a prostate carcinoma, a B-cell lymphoma, a synovial sarcoma of the lung, an adrenal gland carcinoma, a malignant solitary fibrous cancer, and two cancers of unknown primary (CUP). Forty-one of 71 patients with primary tumors were included. One hundred sixty-four metastatic lesions were found in 43 patients.

### Biodistribution in normal organs

**Fig. 1:**
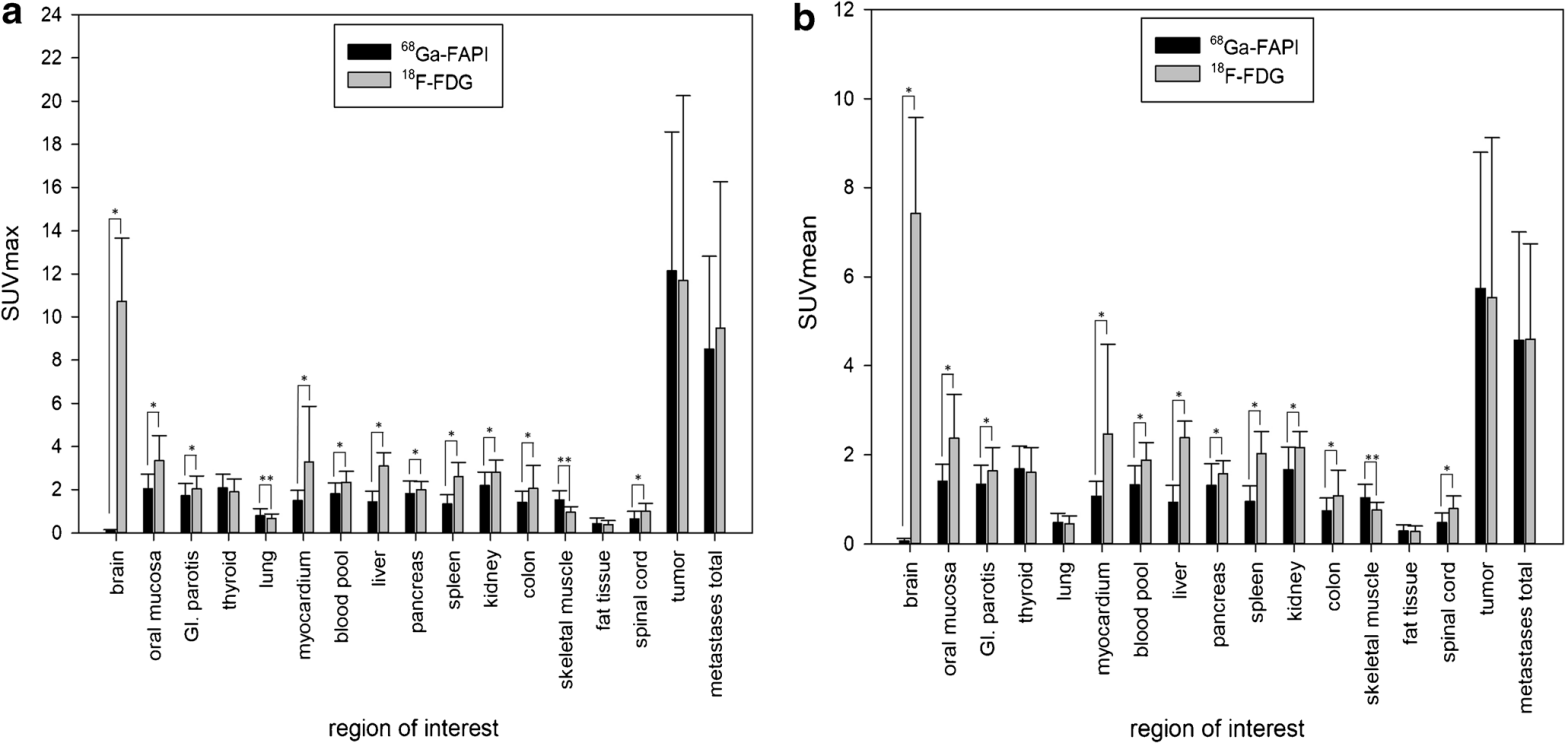
Biodistribution (SUVmax and SUVmean) of ^68^Ga-FAPI in comparison to ^18^F-FDG in normal organs and tumor lesions (mean values and standard deviations; *: ^68^Ga-FAPI sign, higher; **: ^18^F-FDG sign, higher)

**Fig. 2:**
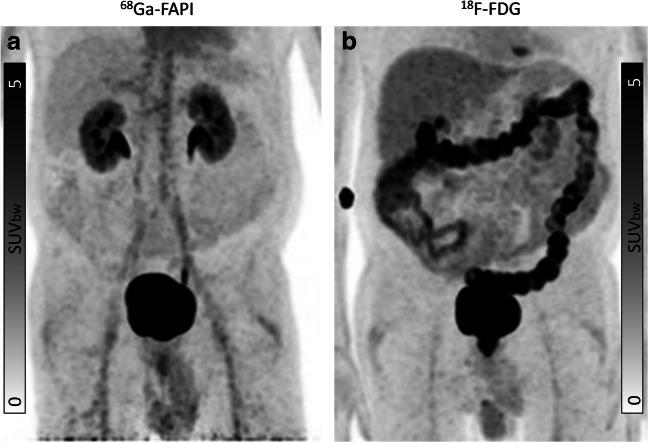
Intra-individual comparison of ^18^F-FDG and ^68^Ga-FAPI in a patient with oral squamous cell carcinoma presenting with inflammation in the colon as incidental finding in the ^18^F-FDG PET/CT (**b**), while no acute inflammatory process is associated with a ^68^Ga-FAPI-positive PET signal (**a**). The direct quantitative comparison presents a high SUVmax of 8.11 in the ^18^F-FDG PET/CT, while ^68^Ga-FAPI PET/CT demonstrates low tracer accumulation (SUVmax: 0.38)

Biodistribution of normal organs for ^68^Ga-FAPI and ^18^F-FDG is presented in Fig. 1. The SUVmean, SUVmax, and TBRs are provided in Supplemental Table 3a, b. ^68^Ga-FAPI uptake was lower than ^18^F-FDG uptake in most normal tissues (11 of 15 organs). Mean SUVmax was significantly lower for ^68^Ga-FAPI than ^18^F-FDG in brain parenchyma (^68^Ga-FAPI vs. ^18^F-FDG: 0.09 vs. 10.72; *p* < 0.001), oral mucosa (2.04 vs. 3.33; *p* < 0.001), parotid gland (1.71 vs. 2.04; *p* < 0.001), myocardium (1.50 vs. 3.27; *p* < 0.001), blood pool (1.81 vs. 2.34; p < 0.001), liver (1.42 vs. 3.10; *p* < 0.001), pancreas (1.82 vs. 1.99; *p* = 0.027), spleen (1.33 vs. 2.60; *p* < 0.001), and kidney cortex (2.20 vs. 2.80; *p* < 0.001). Furthermore, the GI tract showed no relevant ^68^Ga-FAPI uptake as compared to ^18^F-FDG (measured in colon transversum: mean SUVmax 1.40 vs. 2.05; *p* < 0.001; SUVmean 0.74 vs. 1.08; *p* < 0.001) (Fig. 2**)**. No significant difference between ^68^Ga-FAPI and ^18^F-FDG was found in normal fat tissue (SUVmax 0.44 vs. 0.39; *p* = 0.057). Moreover, no significant difference was found in the SUVmean in lung parenchyma (SUVmean 0.48 vs. 0.46; *p* = 0.056) and thyroid tissue (SUVmean 1.68 vs. 1.61; *p* = 0.306), whereas the SUVmax of lung parenchyma (SUVmax 0.79 vs. 0.66; *p* < 0.001) and thyroid tissue (SUVmax 2.08 vs. 1.90; *p* = 0.049) as well as skeletal muscles SUVmean und SUVmax (SUVmax 1.50 vs. 0.95; *p* < 0.001) showed significantly higher uptake for ^68^Ga-FAPI than for ^18^F-FDG.

### Uptake in tumor lesions

**Fig. 3:**
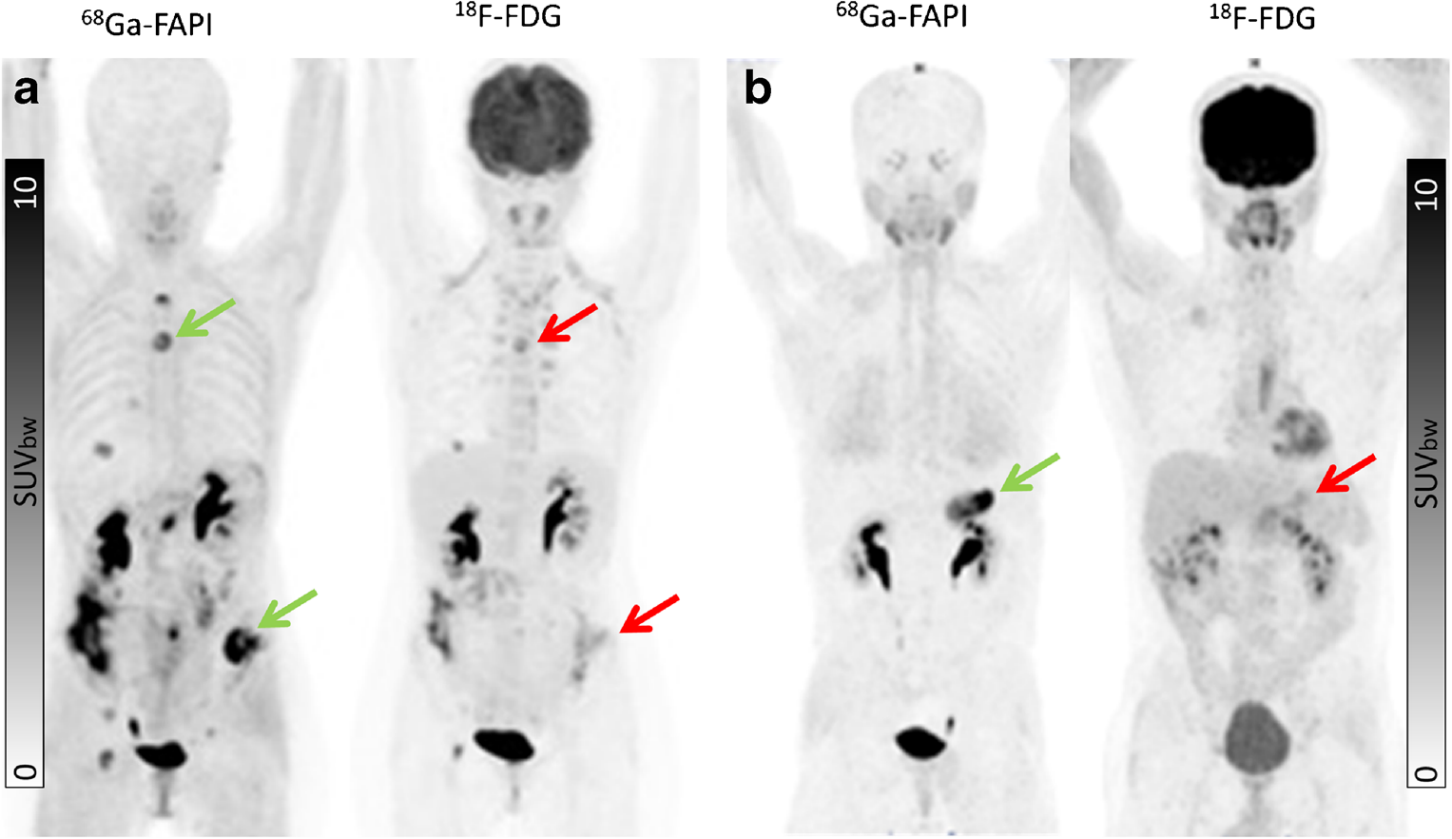
Intra-individual comparison of ^18^F-FDG and ^68^Ga-FAPI in two patients with ovarian cancer (**a**) and pancreas cancer (**b**), respectively. Both present with strong ^68^Ga-FAP uptake in the primary and metastatic lesions while only slight to moderate uptake on ^18^F-FDG PET/CT (arrow: green (^68^Ga-FAPI) and red (^18^F-FDG)

No significant difference in mean SUVmax of ^68^Ga-FAPI and ^18^F-FDG was present in primary tumors (*n* = 41; ^68^Ga-FAPI vs. ^18^F-FDG: 12.14 vs. 11.69; *p* = 0.429) or metastatic lesions in total (SUVmax 8.49 vs. 9.48; *p* = 0.814). The site of metastasis had no significant impact on uptake of ^18^F-FDG or ^68^Ga-FAPI. Mean SUVmax of ^68^Ga-FAPI vs. ^18^F-FDG was 7.89 vs. 11.17; *p* = 0.334 for lymph node metastases, 9.82 vs. 8.84; *p* = 1.000 for liver metastases, 7.83 vs. 7.46; *p* = 0.542 for bone metastases, 6.68 vs. 11.48; *p* = 0.641 for lung metastases and 10.67 vs. 8.17; and *p* = 0.119 for other types of metastases (pleural, peritoneal and soft tissue metastases), respectively. However, in individual patients, marked differences between ^18^F-FDG and ^68^Ga-FAPI tumor uptake were observed. An example of a patient with tumor lesions with low uptake on ^18^F-FDG PET but high uptake on ^68^Ga-FAPI scan is shown in Fig. 3.

### Tumor-to-background ratios

TBRs for lymph node metastases with fatty tissue as background (^68^Ga-FAPI vs. ^18^F-FDG 17.4 vs. 24.8; *p* = 0.132) were the same as were lung metastases to pulmonary parenchyma as background (7.21 vs. 11.31; *p* = 0.313) (Table 1). In contrast, TBRs of bone metastases to bone spongiosa (^68^Ga-FAPI vs. ^18^F-FDG, 7.2 vs. 3.3; *p* = 0.033) and hepatic metastases to liver parenchyma (^68^Ga-FAPI vs. ^18^F-FDG, 5.8 vs. 2.6; *p* = 0.011) were substantially higher with ^68^Ga-FAPI than ^18^F-FDG (Table 1). Primary tumor TBRs were approximately equal between the two agents (Table 2).

**Table 1:** TBRs for different metastases (Lymph node, Bone, Liver, Lung)

VOI	Lymph node metastases/fat tissue (*n* = 26)		Bone metastases/bone spongiosa (*n* = 13)		Hepatic metastases/liver parenchyma (*n* = 14)		Lung metastases/lung parenchyma (*n* = 8)	
Tracer	^68^Ga-FAPI	^18^F-FDG	^68^Ga-FAPI	^18^F-FDG	^68^Ga-FAPI	^18^F-FDG	^68^Ga-FAPI	^18^F-FDG
TBR	17.38	24.76	7.16	3.25	5.84	2.62	7.21	11.31
*p* value	0.132		0.033		0.011		0.313

**Table 2:** Primary tumor TBRs

VOI	Tumor/blood pool		Tumor/skeletal muscle		Tumor/fat tissue	
Tracer	^68^Ga-FAPI	^18^F-FDG	^68^Ga-FAPI	^18^F-FDG	^68^Ga-FAPI	^18^F-FDG
TBR	6.01	3.57	6.99	9.48	27.69	23.96
*p* value	0.002		< 0.001		0.331	

## Discussion

This is an international multicenter retrospective analysis for the intra-individual comparison of ^18^F-FDG with newly developed ^68^Ga-labeled FAP inhibitors [4, 5]. Regardless of the various equipment and imaging protocols used, we report high tumor-to-background ratios for ^68^Ga-FAPI, comparable or superior to ^18^F-FDG that may result in high diagnostic performance for cancer staging and restaging.

**Fig. 4:**
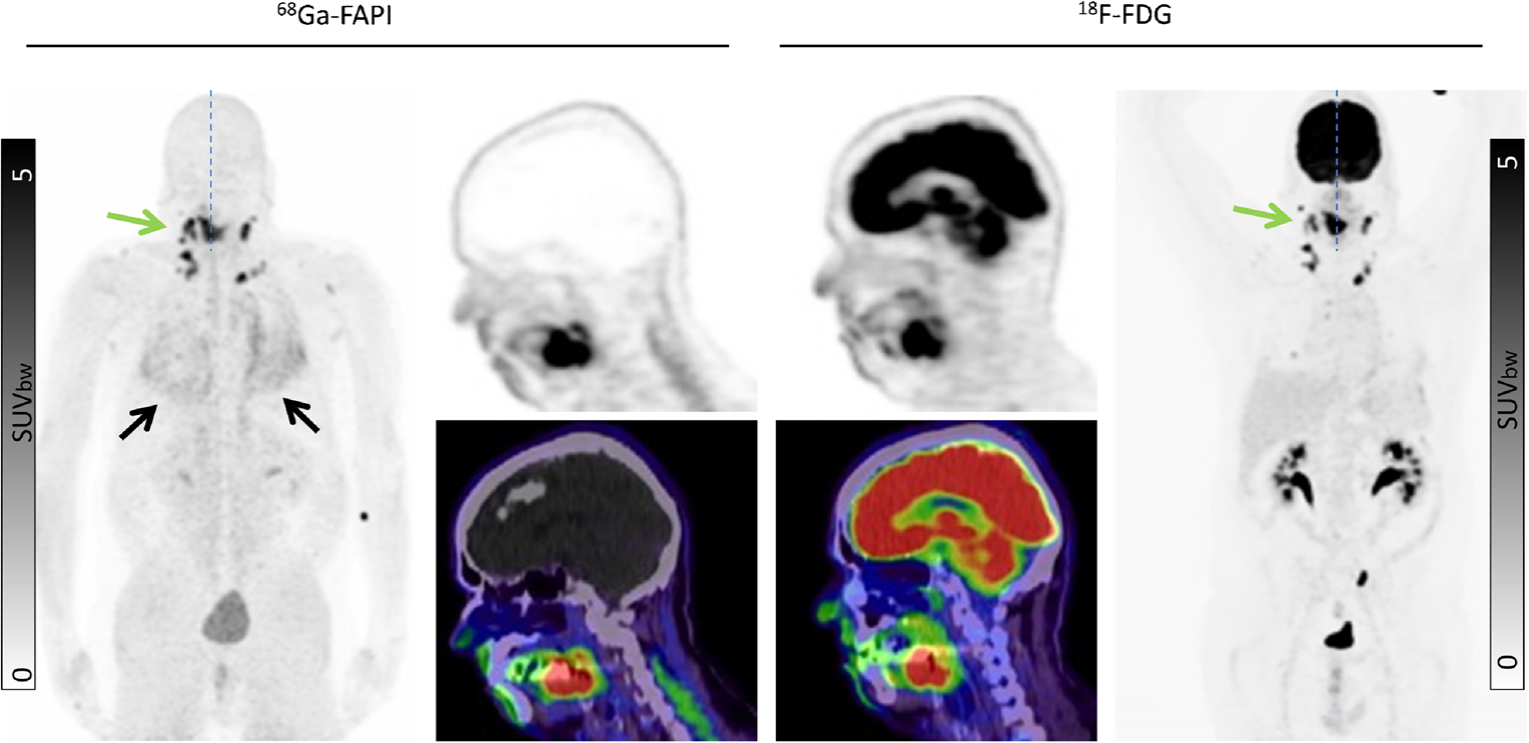
A 68-year-old patient with a histologically confirmed squamous cell carcinoma of the edge of the tongue for pre-radiotherapeutic staging using PET/CT. The quantified uptake in the primary tumor (green arrow) on ^68^Ga-FAPI was SUVmax 20.26 compared to the ^18^F-FDG uptake with an SUVmax 13.35. As a secondary finding, fibrotic, scarred changes of the lung indicative of pulmonary fibrosis were observed (black arrow)

**Fig. 5:**
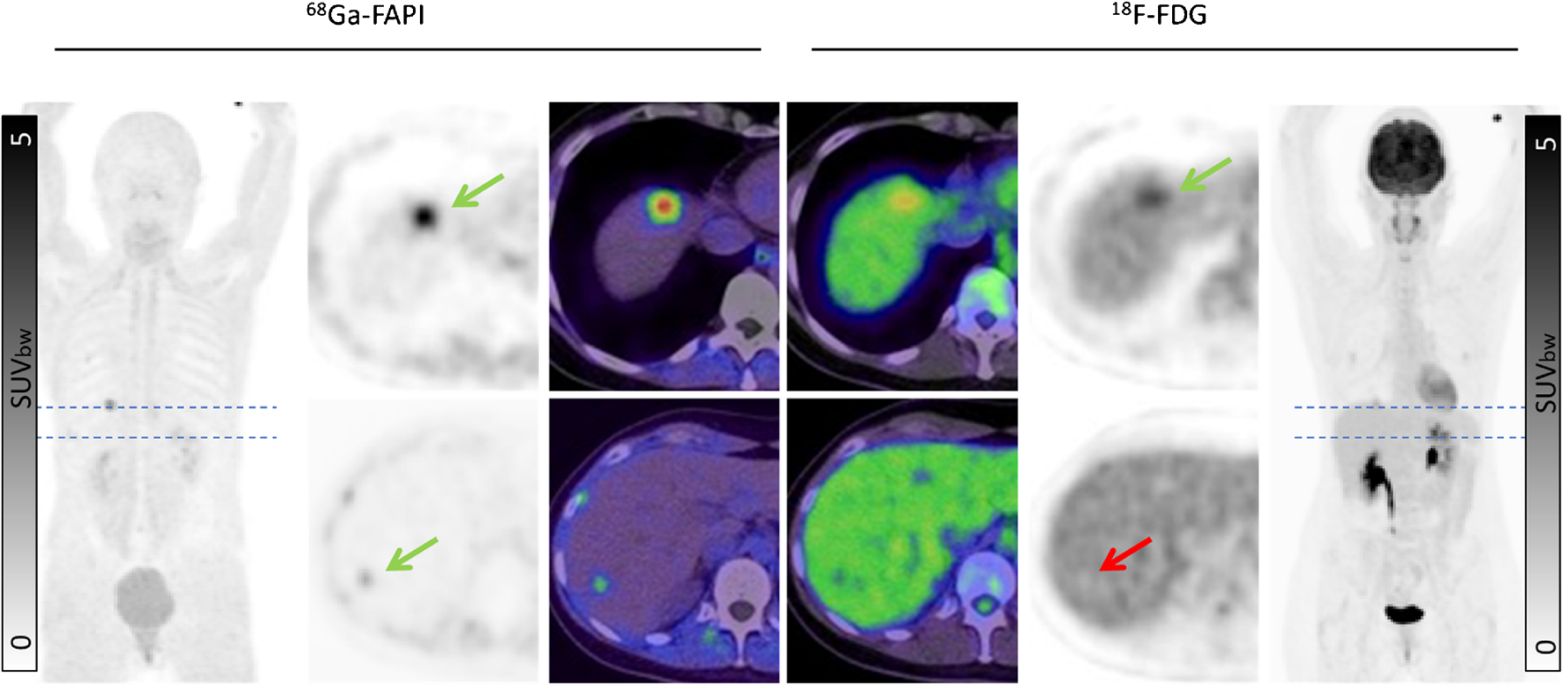
A 40-year-old female patient with ovarian cancer underwent restaging due to a suspicious subdiaphragmatic hepatic lesion. Tracer uptake in the normal liver parenchyma was markedly different on the two tracers: ^68^Ga-FAPI SUVmax 0.79 vs. ^18^F-FDG SUVmax 2.69. In the liver segment III and VII, a strong ^68^Ga-FAP uptake was found (green arrow) compared to ^18^F-FDG. The liver metastatic lesion in segment VII was not detected by ^18^F-FDG (red arrow)

**Fig. 6:**
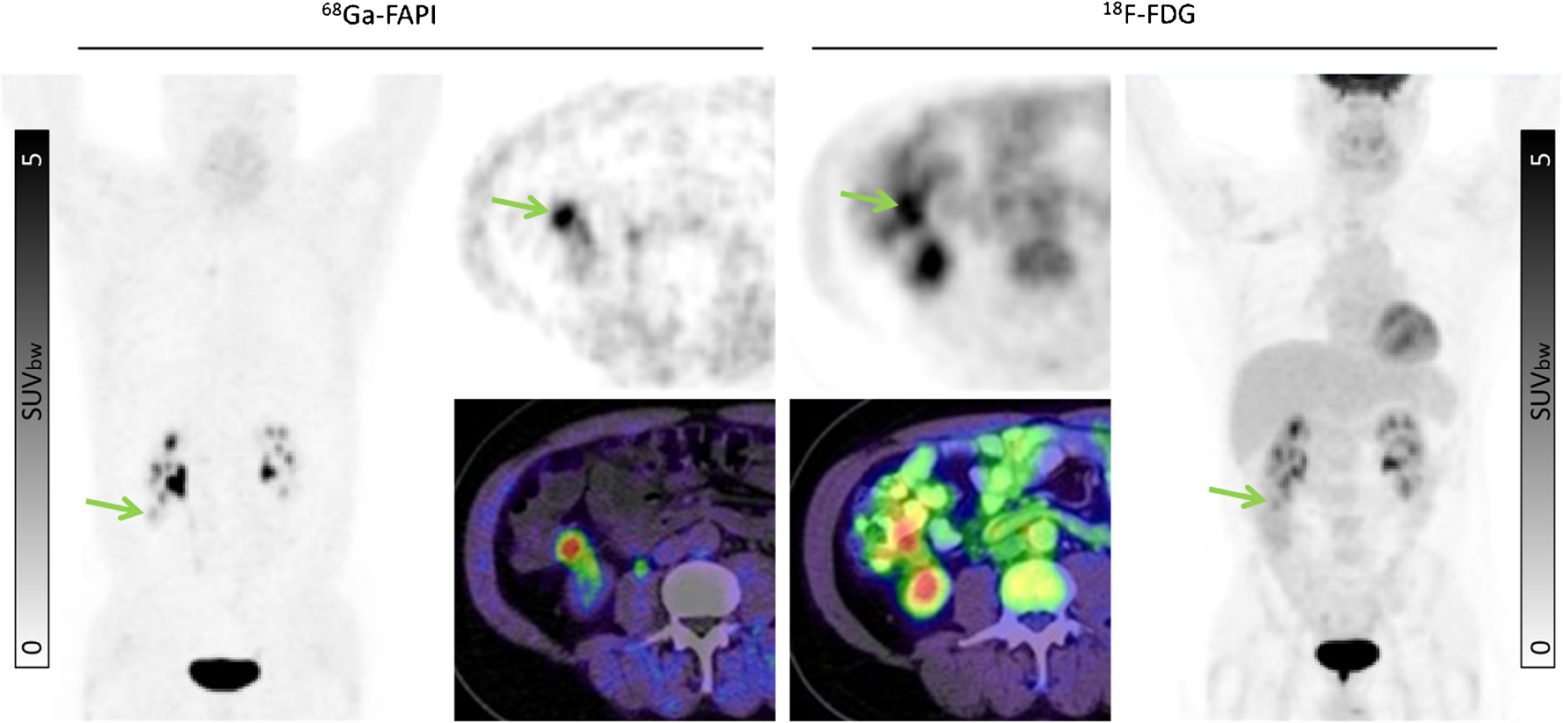
A 55-year-old male underwent pre-operative staging after being diagnosed with colon cancer in the right ascending colon (green arrow). Both tracers presented uptake in the primary tumor (SUVmax: ^68^Ga-FAPI 6.25 vs. ^18^F-FDG 5.34), but there is considerably more background signal on ^18^F-FDG

**Fig. 7:**
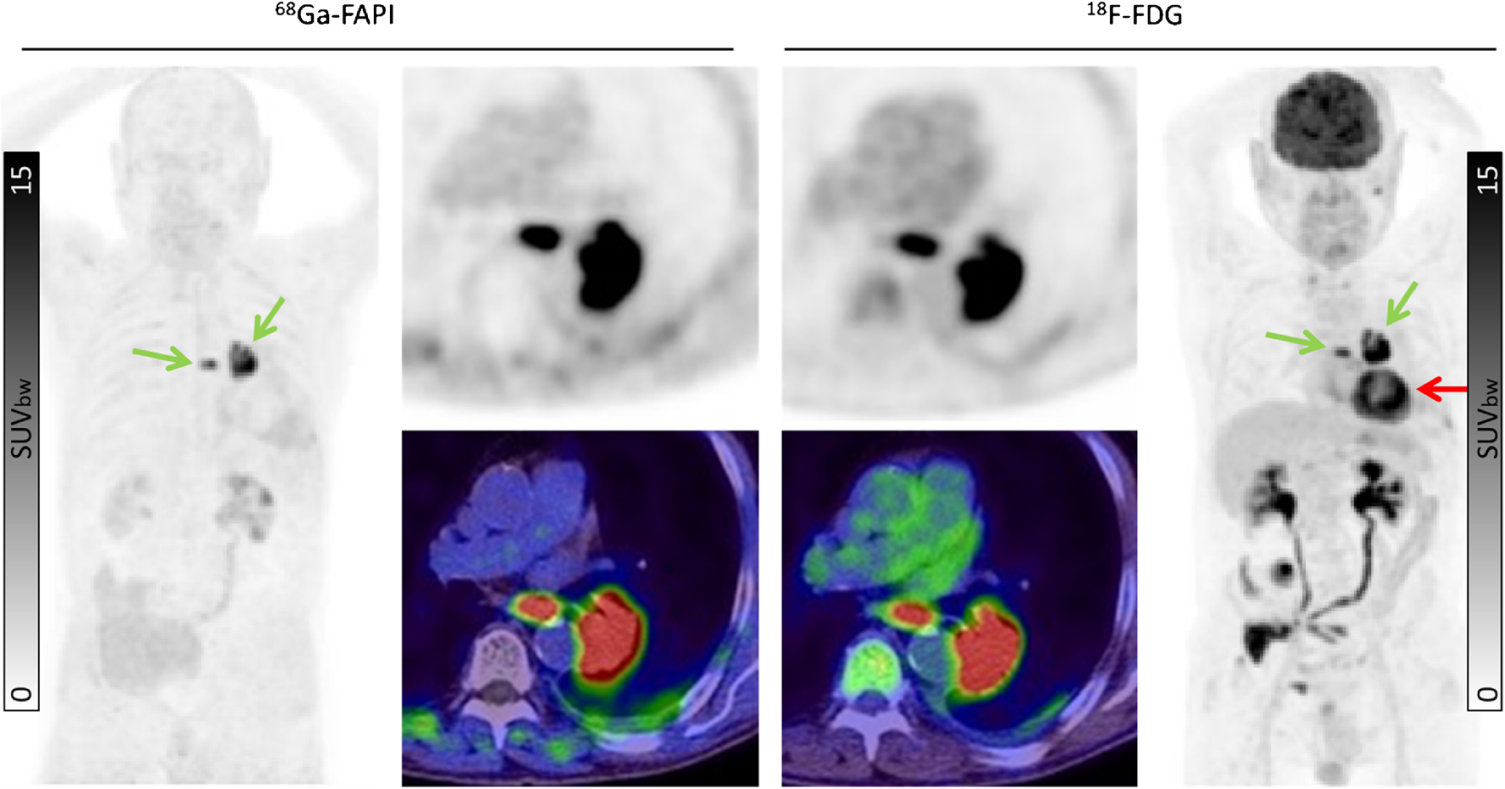
An 80-year-old male patient with lung cancer (green arrows) was diagnosed by ^18^F-FDG PET/CT. ^68^Ga-FAPI PET/CT demonstrated similar tracer uptake (SUVmax: ^18^F-FDG 15.99 vs. ^68^Ga-FAPI 17.95). One advantage of ^68^Ga-FAPI in this instance is the lack of cardiac muscle uptake which is prominent with ^18^F-FDG (red arrow)

Our findings suggest that ^68^Ga-FAPI may improve tumor delineation in organs with physiologically high ^8^F-FDG background activity. This may be relevant in cancers such as pancreatic, ovarian, head-and-neck, hepatocellular and cholangiocarcinoma, colon cancer, as well as brain tumors and lung cancer [12–15]. Due to low ^68^Ga-FAPI uptake in most normal parenchyma, favorable tumor delineation was found in head-and-neck region (Fig. 4), liver metastases (Fig. 5), and abdominal cancer (Fig. 6). Furthermore, in the mediastinal region, ^68^Ga-FAPI uptake in the cardiac muscle is very low compared to ^18^F-FDG (Fig. 7). However, we also examined a diffuse large B-cell lymphoma which showed relatively low ^68^Ga-FAPI uptake. FAP-negative tumor phenotypes have also previously been described in the literature, e.g., in differentiated thyroid carcinoma and renal cell cancer [16–18]. Unfortunately, in this work, we can only report the mean values over a relatively heterogenic group of patients. Further research will be needed to explore dedicated kinds of tumor where staging per FAPI PET might provide a clinical advantage in comparison to FDG PET. In addition to tumor entity, it would also be interesting whether FAPI uptake depends on tumor aggressiveness or proliferation rate as it is for ^18^F-FDG. However, due to small sample sizes within the respective histopathological subtypes, we could not reasonably perform a correlation between grading and FAPI uptake, yet. This investigation would have to be carried out in a future evaluation.

According to previous reports about their respective biodistribution and pharmacokinetics, the ligands FAPI-02, FAPI-04, and FAPI-46 do not differ in early uptake phase 10 min to 1 h p.i. and were thus considered widely exchangeable with regard to our diagnostic evaluation. In comparison to FAPI-02, the ligands FAPI-04 and FAPI-46 have longer tumor retention time beyond 1 h p.i., which however would only be relevant in the perspective of FAP-targeted radionuclide therapy [18, 19]. FAPI-74 either can be used as ^68^Ga-FAPI-74 (this work) or alternatively could also be labeled with ^18^F providing an advantage in large batch production. However, biodistribution at 1 h p.i. was reported comparable to previous ligands [20].

^68^Ga-FAPI and ^18^F-FDG reflect two different aspects of tumor behavior. ^18^F-FDG directly targets tumor cell metabolism. In contrast, ^68^Ga-FAPI targets FAP on CAFs in the stroma of tumors, which is an indicator of desmoplastic reaction and was reported to be one of the key determinants of tumor immunity [19] and multidrug resistance [21] possibly related to reductions in transtumoral transport of cells and drugs. Thus, in addition to its function as a staging modality, FAPI PET/CT may be helpful in understanding tumor biology related to the tumor microenvironment.

Due to the limited number of studies to date, the tumor entities with low or high ^68^Ga-FAPI uptake are also not yet sufficiently known.

Furthermore, FAPI could also play a role in the examination of myocardial infarction and IgG4-related diseases [22, 23].

Our retrospective analysis has several limitations: ^68^Ga-FAPI PET/CT was performed with four different ligands, which however share a common backbone and their early phase biodistribution as well as tumor uptake are comparable [18–20]. Lacking gold standard validation of discrepant lesions, we are not able to evaluate the respective sensitivity, specificity, and accuracy of ^18^F-FDG and ^68^Ga-FAPI, which was beyond the scope of the current work but has already been addressed by other researchers [24].

The main limitation is the long (up to 3 month) interval between the examinations, which may cause “interval progression” that might be non-neglectable in aggressive cancers. However, we had to find a good tradeoff between test vs. re-test reliability and inclusion of a sufficient large patient cohort. For future clinical trials, more stringent inclusion criteria—and alternating order of tracer administration—should are encouraged.

## Conclusion

In this international multicenter retrospective analysis, 71 patients with various cancers underwent both ^18^F-FDG and ^68^Ga-labeled FAP inhibitors. ^68^Ga-FAPI showed equal or higher TBR at lower radiation doses than ^18^F-FDG. These findings suggest that ^68^Ga-FAPI may demonstrate higher diagnostic performance for cancer staging and restaging in various indications. In addition, the high target to background ratios and the low uptake in normal organs also suggest potential use of FAPI ligands as a potential means of treating tumors with therapeutic radioisotopes.

## Supplementary information

ESM 1 (DOCX 48 kb)
