# Supplementary material for: Head-to-head intra-individual comparison of biodistribution and tumor uptake of 68Ga-FAPI and 18F-FDG PET/CT in cancer patients
Source: Eur J Nucl Med Mol Imaging. 2021 Jun 17;48(13):4377–85. doi: 10.1007/s00259-021-05307-1 (PMC8566651; doi:10.1007/s00259-021-05307-1)
Supplement: Supplementary file 1 — (DOCX 48 kb) [file 259_2021_5307_MOESM1_ESM.docx]

**Supplement Table 1**

| **Patients no.** | **Centre*** | **Age** | **Sex** | **Tracer** | **^68^Ga-FAPI MBq** | **^18^F-FDG MBq** | **Diagnosis** | **Time interval (days)** |
| --- | --- | --- | --- | --- | --- | --- | --- | --- |
| **1** | UKHD | **58** | w | ^68^Ga-FAPI-2 | 126 | 299 | Gastrointestinal cancer | 4 |
| **2** | UKHD | **31** | m | ^68^Ga-FAPI-2 | 307 | 344 | Pancreatic biliary cancer | 1 |
| **3** | UKHD | **55** | m | ^68^Ga-FAPI-2 | 298 | 298 | Gastrointestinal cancer | 9 |
| **4** | UKHD | **80** | m | ^68^Ga-FAPI-4 | 308 | 289 | Lung cancer | 19 |
| **5** | UKHD | **53** | m | ^68^Ga-FAPI-4 | 260 | 252 | Pancreatic biliary cancer | 36 |
| **6** | UKHD | **44** | w | ^68^Ga-FAPI-4 | 284 | 269 | Gynecological cancer | 4 |
| **7** | UKHD | **60** | m | ^68^Ga-FAPI-2 | 325 | 321 | Head and neck cancer | 27 |
| **8** | UKHD | **65** | m | ^68^Ga-FAPI-4 | 255 | 330 | Lung cancer | 70 |
| **9** | UKHD | **81** | m | ^68^Ga-FAPI-2 | 222 | 358 | Lung cancer | 1 |
| **10** | UKHD | **40** | w | ^68^Ga-FAPI-4 | 258 | 340 | Gynecological cancer | 7 |
| **11** | UKHD | **77** | m | ^68^Ga-FAPI-2 | 225 | 362 | Head and neck cancer | 6 |
| **12** | UKHD | **65** | m | ^68^Ga-FAPI-4 | 243 | 273 | Pancreatic biliary cancer | 14 |
| **13** | UKHD | **68** | m | ^68^Ga-FAPI-4 | 255 | 309 | Head and neck cancer | 1 |
| **14** | UKHD | **30** | w | ^68^Ga-FAPI-46 | 251 | 300 | Gynecological cancer | 20 |
| **15** | UKHD | **60** | m | ^68^Ga-FAPI-4 | 202 | 334 | Head and neck cancer | 8 |
| **16** | UKHD | **65** | w | ^68^Ga-FAPI-4 | 222 | 323 | Gynecological cancer | 2 |
| **17** | UKHD | **79** | m | ^68^Ga-FAPI-4 | 257 | 320 | Lung cancer | 53 |
| **18** | UKHD | **74** | m | ^68^Ga-FAPI-4 | 225 | 313 | Pancreatic biliary cancer | 13 |
| **19** | UKHD | **48** | m | ^68^Ga-FAPI-4 | 270 | 362 | Head and neck cancer | 53 |
| **20** | UKHD | **50** | w | ^68^Ga-FAPI-46 | 219 | 333 | Lung cancer | 6 |
| **21** | UKHD | **48** | w | ^68^Ga-FAPI-46 | 233 | 299 | Gastrointestinal cancer | 5 |
| **22** | UKHD | **46** | w | ^68^Ga-FAPI-46 | 274 | 309 | Gastrointestinal cancer | 13 |
| **23** | UKHD | **51** | w | ^68^Ga-FAPI-46 | 255 | 288 | Gynecological cancer | 18 |
| **24** | UKHD | **49** | w | ^68^Ga-FAPI-46 | 126 | 310 | Gynecological cancer | 36 |
| **25** | UKHD | **63** | m | ^68^Ga-FAPI-4 | 165 | 315 | Gastrointestinal cancer | 42 |
| **26** | UKHD | **67** | m | ^68^Ga-FAPI-4 | 182 | 291 | Pancreatic biliary cancer | 32 |
| **27** | UKHD | **55** | m | ^68^Ga-FAPI-46 | 247 | 293 | Gastrointestinal cancer | 7 |
| **28** | UKHD | **77** | m | ^68^Ga-FAPI-46 | 263 | 310 | Other cancer | 3 |
| **29** | UHE | **-** | m | ^68^Ga-FAPI-4 | 142 | 346 | Pancreatic biliary cancer | 2 |
| **30** | UKW | **56** | w | ^68^Ga-FAPI-4 | 134 | 291 | Head and neck cancer | 2 |
| **31** | UKW | **60** | m | ^68^Ga-FAPI-4 | 147 | 299 | Head and neck cancer | 16 |
| **32** | UKW | **53** | m | ^68^Ga-FAPI-4 | 150 | 321 | Gastrointestinal cancer | 14 |
| **33** | UKW | **49** | m | ^68^Ga-FAPI-4 | 120 | 294 | Head and neck cancer | 4 |
| **34** | UKW | **74** | m | ^68^Ga-FAPI-4 | 157 | 278 | Head and neck cancer | 2 |
| **35** | UKW | **66** | m | ^68^Ga-FAPI-4 | 115 | 303 | Head and neck cancer | 2 |
| **36** | UKW | **48** | m | ^68^Ga-FAPI-4 | 155 | 296 | Pancreatic biliary cancer | 1 |
| **37** | UKW | **64** | m | ^68^Ga-FAPI-4 | 168 | 300 | Head and neck cancer | 5 |
| **38** | UKW | **78** | m | ^68^Ga-FAPI-4 | 142 | 299 | Other cancer | 5 |
| **39** | UKW | **55** | m | ^68^Ga-FAPI-4 | 158 | 316 | Gastrointestinal cancer | 14 |
| **40** | UKW | **62** | m | ^68^Ga-FAPI-4 | 149 | 327 | Pancreatic biliary cancer | 10 |
| **41** | UKW | **54** | m | ^68^Ga-FAPI-4 | 155 | 317 | Head and neck cancer | 3 |
| **42** | UKW | **45** | m | ^68^Ga-FAPI-4 | 148 | 297 | Pancreatic biliary cancer | 18 |
| **43** | UKW | **60** | m | ^68^Ga-FAPI-4 | 148 | 289 | Pancreatic biliary cancer | 49 |
| **44** | UKW | **40** | w | ^68^Ga-FAPI-4 | 137 | 290 | Other cancer | 21 |
| **45** | UKW | **61** | w | ^68^Ga-FAPI-4 | 125 | 315 | Gastrointestinal cancer | 3 |
| **46** | UCLA | **51** | m | ^68^Ga-FAPI-46 | 185 | 407 | Head and neck cancer | 14 |
| **47** | UCLA | **69** | w | ^68^Ga-FAPI-46 | 185 | 503,2 | Gastrointestinal cancer | 10 |
| **48** | UCLA | **57** | m | ^68^Ga-FAPI-46 | 184,26 | 407 | Other cancer | 23 |
| **49** | UCLA | **69** | w | ^68^Ga-FAPI-46 | 185 | 673,4 | Head and neck cancer | 47 |
| **50** | UCLA | **76** | m | ^68^Ga-FAPI-46 | 185 | 384,8 | Gastrointestinal cancer | 8 |
| **51** | UCLA | **55** | m | ^68^Ga-FAPI-46 | 173,9 | 407 | Gastrointestinal cancer | 40 |
| **52** | UCLA | **36** | w | ^68^Ga-FAPI-46 | 183,89 | 370 | Gynecological cancer | 21 |
| **53** | UCLA | **65** | m | ^68^Ga-FAPI-46 | 185 | 499,5 | Pancreatic biliary cancer | 61 |
| **54** | UCLA | **56** | w | ^68^Ga-FAPI-46 | 185 | 374,81 | Gastrointestinal cancer | 15 |
| **55** | UCLA | **65** | w | ^68^Ga-FAPI-46 | 185 | 399,6 | Gynecological cancer | 23 |
| **56** | UCLA | **61** | m | ^68^Ga-FAPI-46 | 148 | 447 | Gastrointestinal cancer | 89 |
| **57** | UCLA | **51** | w | ^68^Ga-FAPI-46 | 185 | 680 | Gynecological cancer | 14 |
| **58** | UCLA | **73** | m | ^68^Ga-FAPI-46 | 185 | 488,4 | Pancreatic biliary cancer | 14 |
| **59** | UCLA | **56** | w | ^68^Ga-FAPI-46 | 185 | 555 | Gastrointestinal cancer | 19 |
| **60** | TUM | **60** | w | ^68^Ga-FAPI-4 | 239 | 258 | Head and neck cancer | 3 |
| **61** | UKHD | **36** | w | ^68^Ga-FAPI-74 | 255 | 295 | Other cancer | 5 |
| **62** | UP | **62** | w | ^68^Ga-FAPI-46 | 51,8 | 318,2 | Gynecological cancer | 1 |
| **63** | UP | **46** | w | ^68^Ga-FAPI-46 | 81,4 | 233,1 | Gastrointestinal cancer | 2 |
| **64** | UP | **54** | m | ^68^Ga-FAPI-46 | 55,5 | 314,5 | Other cancer | 1 |
| **65** | UP | **51** | w | ^68^Ga-FAPI-46 | 111 | 251,6 | Gynecological cancer | 1 |
| **66** | UP | **60** | w | ^68^Ga-FAPI-46 | 92 | 407 | Lung cancer | 1 |
| **67** | UHE | **68** | m | ^68^Ga-FAPI-46 | 142 | 450 | Head and neck cancer | 4 |
| **68** | UHE | **47** | w | ^68^Ga-FAPI-46 | 101 | 300 | Gynecological | 17 |
| **69** | UHE | **79** | m | ^68^Ga-FAPI-46 | 155 | 338 | Lung cancer | 3 |
| **70** | UHE | **63** | m | ^68^Ga-FAPI-46 | 154 | 358 | Lung cancer | 52 |
| **71** | UHE | **79** | w | ^68^Ga-FAPI-46 | 214 | 323 | Lung cancer | 8 |

* UKHD = Heidelberg University Hospital, UHE = Essen University Hospital, UKW = Wuerzburg University Hospital, TUM = Technical University Munich, UCLA = University of California Los Angeles (United States, USA), UP = University of Pretoria (Republic of South Africa, RSA)

**Supplement Table 2**

| Site | Heidelberg University Hospital | Würzburg  University Hospital | Essen  University Hospital | University of California, Los Angeles | Technical  University  Munich | University of Pretoria |
| --- | --- | --- | --- | --- | --- | --- |
| PET/CT scanner | Biograph mCT Flow, Siemens | Biograph mCT, Siemens | Biograph128 mCT, Siemens | Biograph16, Biograph64, mCT, TruePoint, both Siemens;  Discovery IQ, GE | Biograph, mCT,  Siemens | Biograph mCT 40 slice, Siemens |
| Injected activity (MBq), ^68^Ga-FAPI | 239 | 142 | 142 | 183 | 239 | 78 |
| Injected activity (MBq), ^18^F-FDG | 312 | 302 | 346 | 442 | 258 | 305 |
| Time interval (median days) | 9 | 5 | 6 | 20 | 3 | 1 |
| CT reference (mAs) | 30 | modulated | 91 - 98 | average 88 | 120 | 40 - 150 |
| CT peak kilovoltage (keV) | 130 | 120 | 100 | 120 | 120 | 120 |
| CT slice thickness (mm) | 5 | 5 | 4 - 5 | 3,26 - 5 | 5 | 5 |
| CT slice increment (mm) | 3 - 4 | - | 4 - 5 | - | 3-4 | 5 |
| PET reconstruction | OSEM algorithm | OSEM algorithm | OSEM algorithm | OSEM algorithm | OSEM algorithm | OSEM algorithm |
| Iterations | 2 | 3 | 3 | 2 or 6 | 4 | 4 |
| Subsets | 21 | 24 | 21 | 8 - 24 | 8 | 8 |
| Matrix | 200 x 200 | 200 x 200 | 200 x 200 | 168 x 168 - 200 x 200 |  | 200x200 |
| Corrections | Gaussian 5 mm FWHM | Gaussian 2 mm FWHM (TrueX, Siemens) | Gaussian | Gaussian 5 – 6,3 mm FWHM | Gaussian  5mm  FWHM | Gaussian filter applied at FWHM of 5.0mm |

**Supplement Table 3a**

**Biodistribution SUVmean**

|  |  |  | **68Ga-FAPI** | | **18F-FDG** | |
| --- | --- | --- | --- | --- | --- | --- |
| Organ | N | P-value | SUVmean | Standard deviation | SUVmean | Standard deviation |
| Brain | 71 | < 0,001 | 0,07 | 0,05 | 7,42 | 2,16 |
| Oral mucosa | 70 | < 0,001 | 1,41 | 0,38 | 2,37 | 0,99 |
| Parotis (Mean) | 70 | < 0,001 | 1,33 | 0,43 | 1,64 | 0,52 |
| Thyroid (Mean) | 64 | 0,306 | 1,68 | 0,51 | 1,61 | 0,56 |
| Lung (Mean) | 71 | 0,056 | 0,48 | 0,20 | 0,46 | 0,17 |
| Myocardium | 71 | < 0,001 | 1,07 | 0,33 | 2,47 | 2,01 |
| Blood pool | 71 | < 0,001 | 1,34 | 0,43 | 1,88 | 0,39 |
| Liver | 71 | < 0,001 | 0,93 | 0,38 | 2,38 | 0,37 |
| Pancreas | 57 | < 0,001 | 1,31 | 0,49 | 1,57 | 0,30 |
| Spleen | 67 | < 0,001 | 0,96 | 0,35 | 2,03 | 0,50 |
| Kidney(Mean) | 71 | < 0,001 | 1,68 | 0,53 | 2,17 | 0,35 |
| Intestine | 69 | < 0,001 | 0,74 | 0,28 | 1,08 | 0,58 |
| Muscle | 71 | < 0,001 | 1,03 | 0,30 | 0,75 | 0,18 |
| Fat | 71 | 0,360 | 0,30 | 0,14 | 0,28 | 0,12 |
| Spinal canal | 71 | < 0,001 | 0,47 | 0,22 | 0,79 | 0,29 |
| **Lesion type** |  |  |  |  |  |  |
| Primary | 41 | 0,094 | 5,74 | 3,06 | 5,53 | 3,59 |
| Metastases | 43 | 0,814 | 4,56 | 2,45 | 4,59 | 2,15 |
| Lymph node-Metastases | 26 | 0,243 | 4,00 | 1,51 | 4,84 | 2,31 |
| Bone Metastases | 13 | 0,685 | 4,65 | 4,53 | 3,94 | 1,68 |
| Liver Metastases | 14 | 0,241 | 5,39 | 3,43 | 5,29 | 2,54 |
| Lung Metastases | 8 | 0,844 | 3,74 | 1,59 | 4,68 | 3,62 |
| Other Metastases | 14 | 0,068 | 5,97 | 3,04 | 4,55 | 2,10 |

**Table 3b**

**Biodistribution SUVmax**

|  |  |  | **68Ga-FAPI** | | **18F-FDG** | |
| --- | --- | --- | --- | --- | --- | --- |
| Organ | N | P-value | SUVmean | Standard deviation | SUVmean | Standard deviation |
| Brain | 71 | < 0,001 | 0,09 | 0,06 | 10,72 | 2,96 |
| Oral mucosa | 70 | < 0,001 | 2,04 | 0,66 | 3,33 | 1,18 |
| Parotis (Mean) | 70 | < 0,001 | 1,71 | 0,58 | 2,04 | 0,59 |
| Thyroid (Mean) | 64 | 0,049 | 2,08 | 0,63 | 1,90 | 0,60 |
| Lung (Mean) | 71 | < 0,001 | 0,79 | 0,32 | 0,66 | 0,20 |
| Myocardium | 71 | < 0,001 | 1,50 | 0,47 | 3,27 | 2,59 |
| Blood pool | 71 | < 0,001 | 1,81 | 0,50 | 2,34 | 0,51 |
| Liver | 71 | < 0,001 | 1,42 | 0,50 | 3,10 | 0,61 |
| Pancreas | 57 | 0,027 | 1,82 | 0,58 | 1,99 | 0,38 |
| Spleen | 67 | < 0,001 | 1,33 | 0,43 | 2,60 | 0,66 |
| Kidney (Mean) | 71 | < 0,001 | 2,20 | 0,65 | 2,80 | 0,57 |
| Intestine | 69 | < 0,001 | 1,40 | 0,52 | 2,05 | 1,06 |
| Muscle | 71 | < 0,001 | 1,50 | 0,44 | 0,95 | 0,26 |
| Fat | 71 | 0,057 | 0,44 | 0,25 | 0,39 | 0,17 |
| Spinal canal | 71 | < 0,001 | 0,64 | 0,35 | 1,00 | 0,36 |
| **Lesion type** |  |  |  |  |  |  |
| Primary | 41 | 0,429 | 12,14 | 6,43 | 11,69 | 8,56 |
| Metastases | 43 | 0,814 | 8,49 | 4,31 | 9,48 | 6,78 |
| Lymph node-Metastases | 26 | 0,334 | 7,89 | 3,79 | 11,17 | 9,51 |
| Bone Metastases | 13 | 0,542 | 7,83 | 7,31 | 7,46 | 3,86 |
| Liver Metastases | 14 | 1,000 | 9,82 | 6,44 | 8,84 | 5,14 |
| Lung Metastases | 8 | 0,641 | 6,68 | 2,60 | 11,48 | 11,84 |
| Other Metastases | 14 | 0,119 | 10,67 | 4,52 | 8,17 | 3,93 |
